# Utilizing Large Language Models for Enhanced Clinical Trial Matching: A Study on Automation in Patient Screening

**DOI:** 10.7759/cureus.60044

**Published:** 2024-05-10

**Authors:** Jacob Beattie, Sarah Neufeld, Daniel Yang, Christian Chukwuma, Ahmed Gul, Neil Desai, Steve Jiang, Michael Dohopolski

**Affiliations:** 1 Department of Radiation Oncology, University of Texas (UT) Southwestern Medical Center, Dallas, USA

**Keywords:** electronic health records, natural language processing (nlp), public data, artificial intelligence (ai), clinical trial enrollment

## Abstract

Background

Clinical trial matching, essential for advancing medical research, involves detailed screening of potential participants to ensure alignment with specific trial requirements. Research staff face challenges due to the high volume of eligible patients and the complexity of varying eligibility criteria. The traditional manual process, both time-consuming and error-prone, often leads to missed opportunities. Recently, large language models (LLMs), specifically generative pre-trained transformers (GPTs), have become impressive and impactful tools. Utilizing such tools from artificial intelligence (AI) and natural language processing (NLP) may enhance the accuracy and efficiency of this process through automated patient screening against established criteria.

Methods

Utilizing data from the National NLP Clinical Challenges (n2c2) 2018 Challenge, we utilized 202 longitudinal patient records. These records were annotated by medical professionals and evaluated against 13 selection criteria encompassing various health assessments. Our approach involved embedding medical documents into a vector database to determine relevant document sections and then using an LLM (OpenAI's GPT-3.5 Turbo and GPT-4) in tandem with structured and chain-of-thought prompting techniques for systematic document assessment against the criteria. Misclassified criteria were also examined to identify classification challenges.

Results

This study achieved an accuracy of 0.81, sensitivity of 0.80, specificity of 0.82, and a micro F1 score of 0.79 using GPT-3.5 Turbo, and an accuracy of 0.87, sensitivity of 0.85, specificity of 0.89, and micro F1 score of 0.86 using GPT-4. Notably, some criteria in the ground truth appeared mislabeled, an issue we couldn’t explore further due to insufficient label generation guidelines on the website.

Conclusion

Our findings underscore the potential of AI and NLP technologies, including LLMs, in the clinical trial matching process. The study demonstrated strong capabilities in identifying eligible patients and minimizing false inclusions. Such automated systems promise to alleviate the workload of research staff and improve clinical trial enrollment, thus accelerating the process and enhancing the overall feasibility of clinical research. Further work is needed to determine the potential of this approach when implemented on real clinical data.

## Introduction

Clinical trials are essential for advancing medical knowledge and introducing new treatment paradigms. However, many eligible patients miss out on participating in these trials due to a lack of discussion with their treatment teams, a problem rooted in challenges, such as resource scarcity for patient screening [1,2]. Screening is often manual and inefficient, consuming up to 45 minutes per patient and contributing to a 3-9-hour process for a single enrollment [3]. Physicians, nurses, and research staff highlight time constraints and inadequate support as key obstacles [4,5]. This situation contributes to low enrollment rates, leading to the premature closure of studies and limiting the scope of their findings [6]. The advent of automated patient screening technologies may offer a promising solution by facilitating the identification of potential participants and alleviating the workload on research teams, aiming to enhance patient recruitment efficiency and trial outcomes.

Efforts to automate participant selection have shown promise. Applications that perform automatic matching based on genetic biomarkers have seen relative success, and this workflow was able to perform in real-time with improved accuracy [7]. Natural language processing (NLP) has also been used to analyze unstructured data sources like clinical notes. In combination with additional structured data, results from 2015 showed that these techniques could significantly reduce the patient-trial matching workload [8]. However, these approaches have substantial limitations. Methods relying on information extraction techniques fail to interpret semantic relations correctly [8], and several of these older, existing methods that process free text still require manual preprocessing from domain experts [7]. These barriers may prevent a large-scale implementation across a hospital system as they fail to work across all types of criteria or do not fully address clinical research staff shortages.

In recent advancements, NLP techniques and large language models (LLMs) have significantly evolved, showing potential to transform clinical trial eligibility screening. LLMs are able to take large amounts of unstructured text as input, generating a natural language response to any given prompt. The screening process, inherently reliant on interpreting extensive unstructured text, finds a promising solution in NLP and LLMs due to their advanced reasoning and semantic understanding capabilities [9]. Despite their promise, the comprehensive application and in-depth evaluation of LLMs, including GPT-3.5 Turbo [10], GPT-4 [11], and Llama2 [12], for clinical trial patient screening have been sparse [13].

Our research aims to bridge this gap by employing state-of-the-art LLMs to directly analyze unstructured clinical data, thereby determining patient eligibility for clinical trials. Through this endeavor, we aspire to enhance the efficiency and accuracy of identifying eligible trial participants.

## Materials and methods

Data

We utilized the Harvard University National NLP Clinical Challenges (n2c2) 2018 cohort selection challenge dataset. This dataset comprises 288 longitudinal patient records and information regarding 13 selection criteria. These criteria include drug abuse, alcohol abuse, English proficiency, decision-making capacity, history of intra-abdominal surgery, major diabetes-related complications, advanced cardiovascular disease, dietary supplement intake in the past two months (excluding Vitamin D), diagnosis of ketoacidosis in the past year, aspirin use for myocardial infarction prevention, HbA1C values outside the 6.5%-9.5% range, abnormal creatinine levels, and myocardial infarction occurrence within the past six months. Each patient record was annotated by two medical experts, with any discrepancies resolved through adjudication by a researcher in consultation with a physician [14]. Of the 288 patient records in the n2c2 2018 challenge dataset, 202 were made publicly available for training. We utilized 20 patient records for prompt engineering, while the 182 remaining records were reserved for testing.

Indexing and document transformation

To analyze patient records against specific criteria, we employed GPT-3.5 Turbo and GPT-4, mindful of their context length limits of 16,385 and 32,768 tokens, respectively. This limitation necessitated the careful selection of the most pertinent segments from a patient’s electronic health record (EHR), as the full EHR could not be directly processed.

Initially, we transformed the patient EHR documents into a format suitable for querying. Using LlamaIndex, documents were indexed by first partitioning each into 1024-token sections with a 10% overlap to ensure contextual continuity, a process known as chunking. These chunks were then embedded into a vector database using OpenAI’s ada-002 embedding model, creating vector representations for similarity search and retrieval. The embedded chunks and their original text were stored as key-value pairs in a database, facilitating the identification of the top-k chunks most semantically similar to a given query’s embedding. 

Retrieval-augmented generation (RAG) with LlamaIndex

LlamaIndex enhanced GPT-3.5 Turbo and GPT-4’s accuracy by providing targeted context (EHR text chunks) through RAG. We created a retrieval system that selects the top-k relevant text passages for a given query. For each criterion, we set k = 5 and used the LLM prompt as the document retrieval search query. This process yielded the five most relevant chunks of the patient’s EHR.

Criteria eligibility analysis

**Figure 1:**
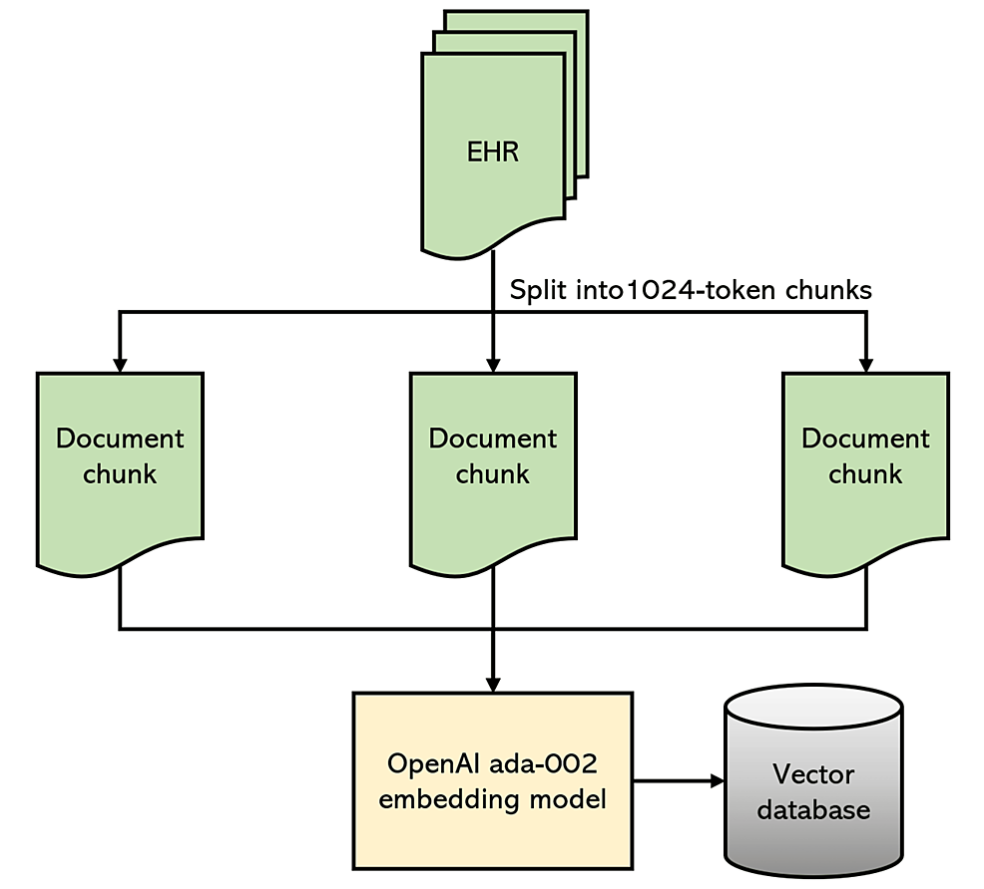
The data indexing process, where unstructured text from patient records is split and embedded into a vector database. EHR: Electronic health record

**Figure 2:**
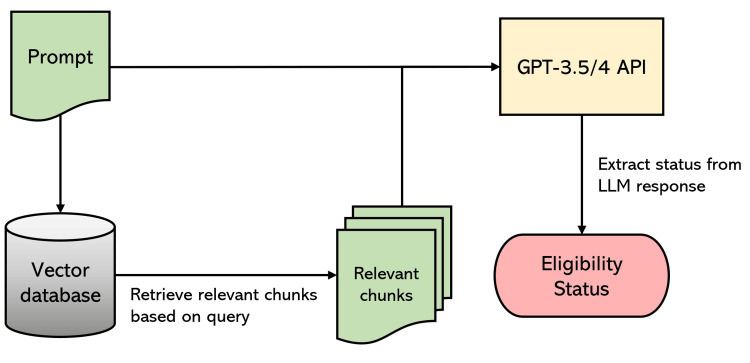
Eligibility screening process for a single patient on a single criterion LLM: Large language model

The relevant EHR portions obtained were appended to the prompt, and GPT-3.5 Turbo was then applied to predict the criteria eligibility status. After processing all five relevant chunks, the final response was saved for analysis. This iterative process of retrieving context and applying the LLM to the prompt/context combination was repeated for each criterion for each patient, culminating in a comprehensive automated eligibility screening depicted in Figures 1, 2.

Prompt engineering

**Figure 3:**
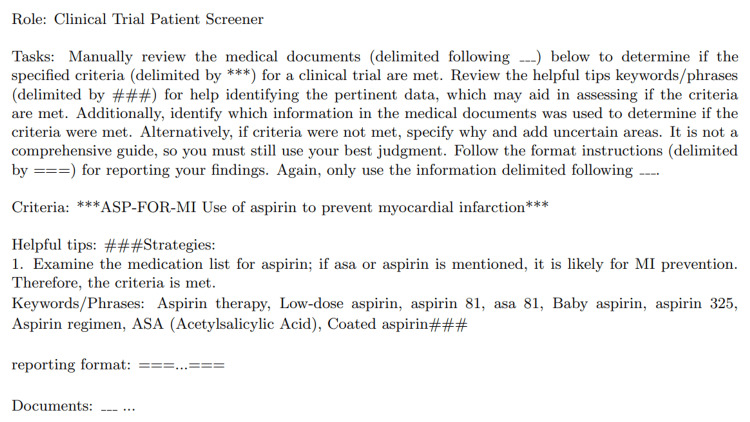
Example prompt for producing JSON output using manually created expert guidance

In our approach, we leveraged the principles of zero-shot learning with GPT-3.5 Turbo and GPT-4, applying a dynamic prompting strategy to evaluate patient eligibility for clinical trials from the n2c2 dataset [15,16]. This strategy utilized a general template, customized with criterion-specific expert guidance and relevant excerpts from patient records to generate tailored prompts for each case (Figure 3). This method combines the zero-shot learning capability of making classifications without direct prior examples of the task with the adaptability of customized prompts, ensuring accuracy and relevance in a clinical context.

The prompt engineering process began with two foundational templates outlining the structure of the inquiry to the LLM: one focused on producing a structured JSON output, and the other focused on producing chain-of-thought (CoT) reasoning. This template was enriched with expert-generated tips to add in targeted patient data retrieval, creating a unique prompt for each eligibility criterion.

To fully automate the screening process, we also created a set of LLM-generated prompts to provide guidance; we used GPT-4 to create criteria descriptions and instructions. These LLM-generated tips consist of vocabulary commonly associated with the criteria being met or not met, emulating rule-based approaches used by the winning team of the original n2c2 challenge [17]. Full prompts for both JSON and CoT output, as well as examples of manual and LLM-created expert tips, can be found in the appendix.

Through iterative testing with a subset of 20 patients, we identified and addressed discrepancies between the LLM’s outputs and the ground truth, refining the manually created expert guidance and prompt templates. This iterative cycle of customization and evaluation continued until we achieved significant accuracy and micro F1 improvements, defined as 0.85, as this was the competitive performance in the n2c2 competition. The two prompting structures were applied to the n2c2 dataset, and their performance was compared. These optimized prompts, embodying the refined integration of expert guidance and patient-specific information, were subsequently applied across the entire dataset. This approach not only harnessed the power of zero-shot learning for efficient patient screening but also enhanced it through tailored prompts, striking a balance between the flexibility of zero-shot learning and the precision needed for clinical applicability.

Analyses

We assessed our model’s performance using accuracy, recall, precision, specificity, and micro F1 metrics across all criteria and patients. Individual criteria were analyzed to highlight specific classification challenges. Our results were benchmarked against the leading teams from the 2018 n2c2 competition to evaluate our standing.

In understanding our model’s limitations, we analyzed misclassifications (false negatives and false positives) by reviewing the LLM’s generated rationales and cross-referencing them with patient charts. This process aimed to identify common error patterns and underlying reasons for inaccuracies, informing future model improvements.

## Results

In our testing encompassing 2366 criteria from 182 patient EHRs from the n2c2 dataset using GPT-3.5 Turbo, we achieved an overall accuracy of 0.81, sensitivity of 0.80, specificity of 0.82, and micro F1 of 0.79. For GPT-3.5 Turbo, the best-performing approach utilized structured JSON output and manually created expert guidance. The full results using GPT-3.5 Turbo are detailed in Table 1. Additionally, we tested our approach on a smaller subset of data (40 patients) due to cost limitations, using GPT-4. Here, we observed an accuracy of 0.87, sensitivity of 0.85, specificity of 0.89, and micro F1 score of 0.86. The full results using GPT-4 are detailed in Table 2. Here, the best-performing approach again utilized structured JSON output and manually created expert guidance. This model’s performance across each criterion is listed in Table 3. Our approach processes a single patient for all included criteria in 1-5 minutes. Variability was due to GPT3.5 and 4 resource allocations during experimentation.

**Table 1:** GPT-3.5 Turbo results when applied to test data (182 patients) CoT: Chain-of-thought; LLM: large language model

Prompting Format	Accuracy	Sensitivity	Specificity	Precision	Micro F1
Structured output + manual expert guidance	0.8081	0.7988	0.8154	0.7721	0.7852
Structured output + LLM-generated expert guidance	0.6593	0.6006	0.7054	0.6148	0.6076
CoT output + manual expert guidance	0.7747	0.6064	0.9066	0.8355	0.7027
CoT output + LLM-generated expert guidance	0.6416	0.3367	0.8802	0.6876	0.4522

**Table 2:** GPT-4 results when applied to a subset of test data (40 patients) CoT: Chain-of-thought; LLM: large language model

Prompting Format	Accuracy	Sensitivity	Specificity	Precision	Micro F1
Structured output + manual expert guidance	0.8692	0.8449	0.8909	0.8734	0.8589
Structured output + LLM-generated expert guidance	0.7712	0.6490	0.8800	0.8281	0.7277
CoT output + manual expert guidance	0.8558	0.8694	0.8436	0.8320	0.8503
CoT output + LLM-generated expert guidance	0.8519	0.8571	0.8473	0.8333	0.8451

**Table 3:** GPT-4 results when utilizing structured JSON output and manually created expert guidance

Criteria	Accuracy	Sensitivity	Specificity	Precision	Micro F1
Drug-Abuse	0.98	0.75	1	1	0.86
Alcohol-Abuse	0.98	1	0.97	0.67	0.80
English	1	1	1	1	1
Makes-Decisions	0.93	0.92	1	1	0.96
Abdominal	0.88	0.71	1	1	0.96
Major-Diabetes	0.83	0.86	0.78	0.83	0.84
ADVANCED-CAD	0.78	1	0	0.78	0.87
DIETSUPP-2MOS	0.70	0.47	0.90	0.82	0.60
KETO-1YR	1	0	1	0	0
ASP-FOR-MI	0.88	0.86	1	1	0.93
HBA1C	0.88	0.58	1	1	0.74
Creatinine	0.85	0.71	0.96	0.92	0.80
MI-6MOS	0.65	0.83	0.62	0.28	0.42
Overall	0.87	0.84	0.89	0.87	0.86

Failure analysis

We performed a thorough failure analysis of the best-performing approach, GPT-4, with structured JSON output and manually created expert guidance. The analysis pinpointed four criteria, DIETSUPP-2MOS, ADVANCED-CAD, and MI-6MOS, with significantly low accuracy rates, with MI-6MOS exhibiting the poorest performance at an accuracy of only 0.65. For MI-6MOS, this suboptimal performance primarily resulted from the LLM’s improper reasoning about dates, leading to several false positives where the LLM recognized myocardial infractions outside of the past six months. The same improper temporal reasoning led to poor performance in DIETSUPP-2MOS, where the LLM categorized patients as meeting the criteria based on dietary supplements they had taken at one point but not within the past two months. For ADVANCED-CAD, the issue was much more nuanced; the LLM correctly identified certain relevant information as per the guidance provided, but the patient did not completely fit all the criteria.

Among all criteria, we observed two prominent types of failures. First, several false negatives were attributed to insufficient document retrieval, where the LLM did not correctly classify a patient as meeting a criterion because no relevant context was provided. Finally, when using GPT-3.5 Turbo, we observed cases of hallucination, with the LLM citing evidence that was either not present or found in the prompt rather than the patient documents. Such hallucinations were observed at a far lower rate in results using GPT-4. For detailed instances of each type of error, see the appendix.

## Discussion

In this study, we have shown that OpenAI’s GPT-3.5 Turbo and GPT-4 offer remarkable zero-shot capabilities for clinical trial eligibility screening, achieving an accuracy of 0.81 and 0.87, respectively, both using the structured output and manually-created expert guidance. Our methodology stands out for its user-friendly nature by requiring little technical knowledge to apply to new criteria, a significant enhancement when compared to traditional screening methods [7,8]. This improvement is marked by an innovative blend of information retrieval and prompting techniques, allowing clinical research staff to utilize natural language for screening criteria fully. This method not only simplifies the screening process, which is predominantly manual in many clinical settings [4] but also drastically reduces the time required for screening from 45 minutes per patient [3] to less than a minute per patient, offering a large efficiency gain.

In the 2018 n2c2 challenge, the Medical University of Graz achieved top results with a micro F1 score of 0.91 [14], using a rule-based system reliant on regular expressions and textual markers [17]. The University of Michigan utilized pattern-based, knowledge-intensive methods to achieve a micro F1 score as high as 0.91 [18], placing second in the original 2018 challenge [14]. However, these methods require significant manual effort, with manual annotation and analysis of 202 patient records enabling such high performance. Furthermore, these approaches necessitate complex technical tools for analyzing natural language, posing a barrier to clinical use. These requirements dramatically limit the accessibility and adaptability of this approach. In contrast, our GPT-3.5 Turbo and GPT-4 approaches offer a more user-friendly and flexible solution capable of interpreting patient data in any supported language without extensive setup or maintenance. Utilizing 10% of available patient records, little to no manual analysis, and little to no technical knowledge from end-users, we achieved competitive results. This adaptability significantly broadens the approach’s application and eases the workload for clinical research teams, highlighting the LLMs’ potential to make clinical trial screening more efficient and inclusive.

Our study marks a significant advancement in applying LLMs, particularly GPT-3.5 Turbo and GPT-4, directly facilitating automatic eligibility screening for clinical trials. This approach represents a departure from existing methods, which either rely on rule-based systems with inherent limitations in flexibility and user-friendliness or necessitate manual preprocessing and struggle to interpret complex semantic relationships in clinical data [7,8]. Unlike previous efforts that utilized LLMs to generate supplemental descriptions for NLP-based models [13], our method leverages LLMs’ advanced semantic analysis capabilities to process unstructured clinical notes directly. This eliminates the need for manual rule definition, preprocessing steps, and additional model training, significantly reducing the operational burden on clinical research staff.

By employing LLMs as the primary engine for screening, we introduce a solution that is more efficient, adaptable, and scalable across different clinical contexts. GPT-3.5 Turbo and GPT-4’s ability to autonomously identify and interpret pertinent information from raw clinical notes streamlines the screening workflow, ensuring contextually aware evaluations without the extensive technical expertise required by traditional methods. This innovation underscores the practical benefits of LLMs in clinical trial screenings, enhancing the process’s efficiency and accuracy while offering a scalable and user-friendly tool for clinical research teams.

In the future, we propose several avenues for improvement. Enhanced prompt engineering techniques, including the adoption of few-shot prompting, offer promising paths to bolster LLM performance directly [9,19,20]. Furthermore, leveraging these prompt engineering techniques when creating LLM-generated expert guidance could potentially increase the screening process’s accuracy. Experimenting with sampling answers from the LLM to ensure decision certainty and expanding our method’s testing across diverse clinical datasets will also be critical in identifying and mitigating weaknesses [21]. Finally, recent developments provide a potential path for customizing prompts on a per-criteria basis automatically, further increasing performance and ease of use [22]. As we refine our approach, our goal remains to balance accuracy with usability, providing clinical research teams with powerful, easy-to-implement tools to revolutionize patient screening processes.

Our approach, while advancing the use of LLMs in clinical trial eligibility screening, has several limitations, including variability in performance across different criteria, particularly with those that are time-sensitive or necessitate multiple requirements to be met. In these cases, our approach can fail to understand certain semantics within the patient EHR, including the relation of different events within a specific patient's medical history. Additionally, the propensity of LLMs to generate incorrect evidence, known as hallucinations, poses a challenge to achieving consistent and reliable results. The possibility of hallucinations is particularly important to note within the medical context when consistency and confidence are incredibly important. These issues highlight the need for further research and refinement to enhance trust and applicability in clinical settings.

## Conclusions

In our study, we leveraged the advanced capabilities of GPT-3.5 Turbo and GPT-4, combined with document retrieval technologies, to innovate patient eligibility screening for clinical trials. This approach, utilizing dynamically generated prompts based on expert guidance and raw clinical data, minimizes manual intervention while offering extensive adaptability across medical disciplines. With its promise of scalability and ease of implementation, our method opens new avenues for enhancing the efficiency and effectiveness of clinical trial screenings.

## Appendices

Prompting

To induce a structured output, we use Pydantic, a data validation library for Python. This allows us to generate a set of formatting instructions automatically. This guides the LLM to produce its output as a JSON object, allowing for easy parsing. As the criteria description, the description and name provided in the original n2c2 challenge were used without modification. The full prompt structure can be seen below. The output will be provided as a JSON object with keys ”criteria name”, ”support”, and ”criteria”, representing the criteria label, cited evidence, and met/not met status respectively

Role: Clinical Trial Patient Screener Tasks: Manually review the medical documents (delimited following ___) below to determine if the specified criteria (delimited by ***) for a clinical trial are met. Review the helpful tips (delimited by ###) for identifying the pertinent data that may help aid screening. It is not a comprehensive guide, so you will still need to use your best judgment; the keywords and phrases can also help. Follow the format instructions (delimited by ===) for reporting your findings. Criteria: ***...*** Helpful tips: ###...### Reporting format: ===The output should be formatted as a JSON instance that conforms to the JSON schema below. As an example, for the schema {"properties": {"foo": {"title": "Foo", "description": "a list of strings", "type": "array", "items": {"type": "string"}}}, "required": ["foo"]} the object {"foo": ["bar", "baz"]} is a well-formatted instance of the schema. The object {"properties": {"foo": ["bar", "baz"]}} is not well-formatted. Here is the output schema: ``` {"properties": {"criteria_name": {"description": "Name of criteria", "title": "Criteria Name", "type": "string"}, "support": {"description": "include the information that supports if criteria is met or not,", "title": "Support", "type": "string"}, "criteria": {"description": "Evaluating if an individual criterion is met after reviewing the medical document. Output should be True or False", "title": "Criteria", "type": "string"}}, "required": ["criteria_name", "support", "criteria"]} ``` === Documents: ___ ...

Due to the potential accuracy benefits of inducing CoT reasoning from a LLM, we use a prompt structure that utilizes CoT reasoning. The LLM is prompted to decide, think step-by-step, and provide its thought process. Then, the LLM is asked to use this reasoning to categorize a criterion as ”met” or ”not met.” In this prompt structure, the n2c2 criteria titles are not included, only the criteria description. The full, prompt structure for ASP-FOR-MI can be seen below. The output is not structured naturally but is saved into a JSON object with the same format as the structured prompt results for easy analysis.

Role: Clinical Trial Patient Screener You are a clinical trial eligibility screener. You have expert medical knowledge and can make definitive judgments on whether criteria are met or not. Specifically, you will be screening for the following criterion: Criteria: ***...*** You will be given a set of documents from a patient's health record. Manually review the medical documents to determine if the above criterion is met or not met. Follow this process when screening for the above criteria: 1. Extract evidence from the medical record to determine if the criterion is met. Use the tips below: ---...--- 2. Think step-by-step. Identify which information in the medical documents was used to determine if the criterion is met. Alternatively, if the criterion was not met, specify why and list uncertain areas. Quote sections of the medical record that support your decision. Assume that the patient is average and able-bodied unless stated otherwise. Consider all documents at once, and should any single document show a criterion that is met, then the criterion is met for the patient. Make a definitive choice, and state clearly if the criterion is met or not. Take a deep breath and analyze the documents below: ___ ...

Expert guidance

Through fine-tuning over 20 patient records, we created a set of expert tips to assist the LLM in the screening process. These tips are inserted directly into the prompt, guiding how to go about the screening process and relevant keywords and phrases. The full, manually created tips for all criteria are listed below.

DRUG-ABUSE: Strategies: 1. Review the social, past medical, and family sections for illicit drug use. 2. Look for rehab, detox, or addiction-related interventions. 3. Check for addiction management meds or multiple controlled substance prescriptions. 4. Scan for overdoses, withdrawal symptoms, or drug-linked incidents. 5. Review drug screenings or relevant toxicology results. 6. Check for counseling on drug use risks or medication misuse. Keywords/Phrases: Opioids: Heroin, Fentanyl, Oxycodone. Stimulants: Cocaine, Methamphetamine. Depressants: Benzodiazepines (e.g., Xanax), Barbiturates. Hallucinogens: LSD, PCP. Cannabinoids: Marijuana, THC. Club Drugs: MDMA, GHB. Others: Inhalants, Steroids, Prescription med abuse. ALCOHOL-ABUSE: Strategies: Female: No more than 1 drink in a single day and no more than 7 drinks per week. Male: No more than 2 drinks in a single day and no more than 14 drinks per week 1. Review clinical notes for mentions of alcohol intake frequency, quantity, or patterns. 2. Check for records of discussions, interventions, or counseling sessions specifically addressing alcohol consumption. 3. Look for physical signs often associated with excessive alcohol use, such as facial flushing, broken blood vessels on the face, or palmar erythema. 4. Monitor liver function tests, GGT levels, and any imaging results revealing liver changes or damage. Keywords/Phrases: Drinking Patterns: Social drinking, Heavy drinking, Daily drinking, Morning drinking. Liver Issues: Fatty liver, Alcoholic liver disease, Ascites, Jaundice. Intervention: AA (Alcoholics Anonymous), Counseling, Alcohol cessation. Complications: Withdrawal symptoms, Delirium tremens, Gastritis, Esophageal varices. Behavioral Indicators: Blackouts, Alcohol-related accidents, DUIs. ENGLISH: Strategies: 1. Check if translation was needed during visits. 2. If not specified, assume English-speaking. ie default answer is True. Keywords/Phrases: English proficiency, non-English speaking, Interpreter used, Fluent in English, ESL. MAKES-DECISIONS: Strategies: 1. If a child, assume cant make their own decisions 2. Review notes on cognitive function and decision-making capacity, if they have limited cognitive funtion or capacity then likely cant make decisions. 3. If medical decision-making is not specified, assume the patient makes their own decisions. ie default answer is True. Keywords/Phrases: Child, Guardian,  Legal guardian, Power of attorney. ABDOMINAL: Strategies: 1. If any of the following surgeries are mentioned in Keywords/Phrases, then the criterion is met. 2. If no surgeries are mentioned, then the criterion is not met. ie answer is False. 3. If minor surgery like cardiac catheterization or stent placement is mentioned, then the criteria are not met. ie answer is False. 4. Surgeries in the chest, neck, head, eyes, legs, or arms are not considered abdominal surgeries. ie answer is False. 5. bypass grafts, angioplasty, and stent placement are not considered abdominal surgeries. ie answer is False. Keywords/Phrases: Laparotomy, Bowel resection, Colectomy, Enterectomy, Abdominal surgery, Hernia repair, Cholecystectomy (gallbladder removal), Appendectomy (appendix removal), Gastrectomy (stomach removal), Splenectomy (spleen removal), Pancreatectomy (pancreas surgery), Cystectomy (bladder removal), Nephrectomy (kidney removal), Lysis of adhesions, bowel obstruction, ileus. MAJOR-DIABETES Strategies: First, the patient must have a history of diabetes, then review for any of the following complications. 1. Review for any signs of foot complications often seen in diabetes, like ulcers, neuropathy, amputation, or poor circulation. If the patient has diabetes and foot complications, then the criterion is met. 2. Review for signs of retinopathy, optic neuropathy, macular edema, or other vision issues related to diabetes. If the patient has diabetes and vision issues, then the criterion is met. Cataracts alone do not meet the criterion 3. Review for signs of kidney damage or nephropathy, like chronic kidney disease, elevated Creatinine, and dialysis. If the patient has diabetes and related kidney disease, then the criterion is met. 4. Diabetic skin conditions, including diabetic dermopathy, necrobiosis, lipoidica, diabeticorum, and ulcers. If the patient has diabetes and similar diabetes-related skin issues, then the criterion is met. Keywords/Phrases: Diabetic foot ulcer, Retinal damage, Neuropathy, optic neuropathy, Nephropathy, Diabetic kidney disease, Peripheral artery disease, Diabetic macular edema, Peripheral neuropathy, Autonomic neuropathy, skin ulcer, chronic kidney disease, CKD, sacral ulcer, decubitus ulcer, foot ulcer, toe ulcer. ADVANCED-CAD Strategies: The patient does not need to match all but just one of the following criteria. Also, note that this is not a comprehensive strategy, so it is to use the best judgment. 1. Review these for a history of CAD, angina, or other heart-related issues. If mentioned, then the criterion is met. 2. Scan for multiple CAD-related medications. Two or more key medications for CAD, such as beta-blockers, calcium channel blockers, or antiplatelet agents (like Plavix, but excluding aspirin). If mentioned, then the criteria are met. 3. Examine results from tests like angiograms, echocardiograms, cardiac catheterization, and stress tests for signs of ischemia or damaged heart tissue. If mentioned, then the criteria are met. 4. Look out for any stent/ placements. If mentioned, then the criterion is met Keywords/Phrases: Myocardial infarction, Stable/unstable angina, Ischemic heart disease, Coronary artery stents, CAD, congestive heart failure, CHF, Coronary artery bypass graft (CABG), Angioplasty, Nitroglycerin, Beta-blockers, Calcium channel blockers, Plavix, Chest discomfort, Echocardiogram findings, cardiac catheterization. Drugs that could be mentioned include Aspirin, Clopidogrel (Plavix), Ticagrelor (Brilinta), Prasugrel (Effient), Warfarin (Coumadin), Rivaroxaban (Xarelto), Apixaban (Eliquis), Metoprolol (Lopressor, Toprol XL), Atenolol (Tenormin), Bisoprolol (Zebeta), Carvedilol (Coreg), Amlodipine (Norvasc), Diltiazem (Cardizem, Tiazac), Verapamil (Calan, Verelan), Nifedipine (Adalat CC, Procardia), Nitroglycerin (Nitrostat, Nitro-Dur), Isosorbide dinitrate (Isordil), Isosorbide mononitrate, Atorvastatin (Lipitor), Simvastatin (Zocor), Rosuvastatin (Crestor), Pravastatin (Pravachol), Lisinopril (Zestril, Prinivil), Enalapril (Vasotec), Ramipril (Altace), Losartan (Cozaar), Valsartan (Diovan), Spironolactone (Aldactone), Eplerenone (Inspra), Ranolazine (Ranexa), Digoxin (Lanoxin), and Ivabradine (Corlanor). DIETSUPP-2MOS Strategies: 1. Review the medical documents for items listed that match the keywords/phrases below. If so, the criteria is met. 2. Consider extracting all the medications, then do a step-by-step comparison to the keywords/phrases below. If any match, then the criterion is met. Keywords/Phrases: Not a comprehensive list, so can use your judgment but the list includes - Dietary supplement, Herbal supplement, Multivitamins, Over-the-counter, OTC, Minerals, calcium, vit e, vit a, vitamin e, vitamin a, Amino acids, Probiotics, Fish oil, Omega-3, Herbal tea, Natural remedy, Supplement brand names (e.g., Centrum, Nature's Bounty). Coenzyme Q10, Turmeric/Curcumin, Glucosamine, Chondroitin, Biotin, Melatonin, Zinc, Magnesium, Green tea extract, Echinacea, Ginkgo biloba, St. John's wort, Ginseng, Flaxseed oil, Whey protein, Collagen, Fiber supplements, Lysine, and brand names such as NOW Foods, Garden of Life, and Solgar in your list of supplements. Ashwagandha, Saw Palmetto, Milk Thistle, Elderberry, Cranberry, Lutein, Selenium, Iron, Folate, B-complex vitamins, L-theanine, Creatine, Resveratrol, Moringa, Maca root, Valerian root, Rhodiola, SAM-e, BCAAs (Branched-Chain Amino Acids), Calcium, L-carnitine, green tea, ginger, Chromium, Iodine, Potassium, Spirulina, Bee pollen, Propolis, and brand names such as Pure Encapsulations, Jarrow Formulas, and Bluebonnet Nutrition. KETO-1YR Strategies:
1. Look for any visits related to diabetic crises, with a particular focus on episodes characterized by severe hyperglycemia or the presence of ketones. 2. Examine the clinical presentation, such as dehydration or altered consciousness, which might indicate ketoacidosis. 3. Look for any mentions of insulin non-compliance or instances where insulin might have been omitted intentionally or unintentionally. Keywords/Phrases: Ketoacidosis, Diabetic ketoacidosis (DKA), Hyperglycemic crisis, Blood ketones, Metabolic acidosis,  Blood gas analysis, Ketonuria, kussmaul respiration, insulin non compliance ASP-FOR-MI Strategies: 1. Examine the medical documents for mention of aspirin, If asa or aspirin is mentioned it is likely it is for MI prevention. Therefore, the criterion is met. 2. Unless aspirin is mentioned under "allergies," then criterion is not met Keywords/Phrases: Aspirin, Low-dose aspirin, aspirin 81, asa 81, Baby aspirin, aspirin 325, Aspirin regimen, ASA (Acetylsalicylic Acid), Coated aspirin HBA1C Strategies: Review Chemistry/Laboratory data for all HbA1c levels. 1. if HbA1c is mentioned, check if a value is given. If not, assume it is not between 6.5 and 9.5. The default answer is False. Otherwise 2. Extract the numerical value after the word "HbA1c" or "A1c" and compare it to the normal range. If the value is between 6.5 and 9.5, the criterion is met. 3. If multiple values are given, use the largest value. If no value is given, assume it is not between 6.5 and 9.5. The default answer is False. 4. Sample Example: "HbA1c 7.2%" or "A1c 8.5%" would meet the criterion. But "HbA1c 5.8%" or "A1c 10.1%" would not. 
Keywords/Phrases: Hemoglobin A1c, HbA1c test results, A1c, A1c percentage, Hemoglobin A1c level CREATININE Strategies: A normal result is 0.7 to 1.3 mg/dL (61.9 to 114.9 (*µ*)mol/L) for men and 0.6 to 1.1 mg/dL (53 to 97.2 (*µ*)mol/L) for women. Review all the medical records for mention of creatinine/Cr or other keywords. 1. Extract all numerical values after the word "creatinine" or "Cr" and compare them to the normal range. If any value is higher than the upper limit of normal (1.3 for men and 1.1 for women), the criterion is met. 2. If provided with a range, then take the largest value of that range and analyze it. For example, Cr 1.6-2.0 would be elevated because 2.0 is abnormal 3. If multiple values are given, use the largest value. 4. If no value is given, assume it is not above the upper limit of normal. ie answer is False. Keywords/Phrases: Serum creatinine, Cr, Chemistry, Lab, Creatinine blood test, LABORATORY DATA MI-6MOS Strategies: Needs to be a relatively recent MI 1. Look for reported symptoms of chest pain, shortness of breath, or other signs of a heart attack. However, symptoms alone are not enough to meet the criteria. They will need to have evidence of elevated cardiac biomarkers or ECG changes. 2. If troponin levels are mentioned, check if they are elevated. If so, the criterion is met. 3. Look for recent ECG changes, such as ST-segment elevation or T-wave inversion. If patient had symptoms of a heart attack and ECG changes, the criteria is met. 4. See if procedures like cardiac catheterization or angioplasty were performed or planned. If so, the criterion is met. 5. If STEMI, NSTEMI, or myocardial infarction are mentioned, the criterion is met.
Keywords/Phrases: STEMI, NSTEMI, Myocardial infarction, Recent heart attack, Troponin elevation, Cardiac enzymes, Post-MI changes, Coronary syndrome, Elevated troponin levels, ECG changes, Acute coronary event, stent, cardiac cath.

To fully automate the eligibility screening process, we used LLM's abilities to generate expert guidance without manual intervention. The LLM is provided with the criteria description and tasked with generating vocabulary, keywords, and phrases associated with the criteria being met or not met. This is done to assist with reasoning and the document retrieval process. The full list of LLM-generated expert tips can be found below.

DRUG-ABUSE: Associated with meeting the criterion of evidence of drug abuse, current or past, you might encounter terms such as substance abuse, addiction, positive drug screen, toxicology report, withdrawal symptoms, tolerance, dependency, rehabilitation, relapse, narcotics, stimulants, depressants, hallucinogens, drug-seeking behavior, overdose, and history of substance use disorder. For not meeting the criterion, terms could include a negative drug screen, sobriety, abstinence, clean toxicology report, recovery, non-user, and no history of substance abuse.' 'req': 'Evidence of Drug abuse, current or past ALCOHOL-ABUSE: Associated with meeting the criterion of current alcohol use over weekly recommended limits might include terms such as "heavy drinking," "excessive alcohol consumption," "binge drinking," "alcohol abuse," "high alcohol intake," "above recommended limits," "chronic alcohol use," "frequent intoxication," "alcohol dependence," and "positive alcohol screening test." Conversely, terms indicating the criterion is not met could include "moderate drinking," "within recommended limits," "low-risk alcohol use," "abstinence," "sober," "infrequent drinking," "negative alcohol screening test," "responsible drinking," and "alcohol consumption within guidelines. ENGLISH: Associated with the criterion being met: fluent, proficient, English-speaking, bilingual, native speaker, conversational level, language proficiency, understanding English, communicating in English, and English literacy. Associated with criterion not being met: non-English speaking, limited English, language barrier, requires interpreter, non-fluent, poor comprehension, language difficulty, non-native speaker, ESL (English as a Second Language), inadequate English skills. MAKES-DECISIONS: Associated with the criterion being met: competent, autonomous, decision-making capacity, informed consent, self-determined, cognitive ability, understanding, voluntary, legal age, emancipated minor, mental capacity, lucid, coherent, unimpaired judgment. Associated with the criterion not being met: incapacitated, cognitive impairment, dementia, Alzheimer's, minor, underaged, legally incompetent, guardian, power of attorney, conservatorship, delirium, psychiatric illness, impaired consciousness, coerced, undue influence. ABDOMINAL: Associated words for meeting the criterion of a history of intra-abdominal surgery, small or large intestine resection, or small bowel obstruction might include appendectomy, colectomy, gastrectomy, hysterectomy, laparotomy, cholecystectomy, bowel resection, anastomosis, adhesiolysis, ileostomy, colostomy, diverticulectomy, and enterectomy. Words indicating complications or related conditions could be adhesions, hernia repair, or postoperative ileus. Words indicating the criterion is unmet might include no prior surgery, intact bowel, no history of obstructions, non-surgical management, and uncomplicated abdominal history. Terms like laparoscopy or endoscopy might appear in both contexts but would need clarification, as they could refer to diagnostic procedures without any resection or obstruction. MAJOR-DIABETES: Associated with meeting the criterion of having a history of diabetes and major diabetes-related complications, words might include hyperglycemia, insulin, type 1 diabetes, type 2 diabetes, HbA1c, diabetic neuropathy, retinopathy, diabetic foot ulcer, nephropathy, cardiovascular disease, stroke, myocardial infarction, peripheral artery disease, and end-stage renal disease. Words indicating the criterion is unmet could include normoglycemia, non-diabetic, no history of hyperglycemia, absence of retinopathy, healthy renal function, no neuropathy, intact peripheral circulation, and no cardiovascular complications. These terms suggest the individual does not have diabetes or has not developed significant complications associated with the condition. ADVANCED-CAD: Associated with meeting the criterion of advanced cardiovascular disease: myocardial infarction, congestive heart failure, angina pectoris, coronary artery disease, cardiomyopathy, arrhythmia, peripheral arterial disease, stroke, transient ischemic attack, atherosclerosis, heart valve disease, aneurysm, ischemic heart disease, left ventricular dysfunction, stent, bypass surgery, angioplasty, electrocardiogram abnormalities, echocardiogram abnormalities, heart failure, elevated troponin, elevated BNP (B-type natriuretic peptide), abnormal stress test, pacemaker, implantable cardioverter-defibrillator (ICD), severe hypertension, advanced atheroma. Associated with not meeting the criterion of advanced cardiovascular disease: normal blood pressure, normal cholesterol levels, normal EKG/ECG, normal echocardiogram, no history of cardiac events, no interventions (stents, bypass), no symptoms (chest pain, dyspnea), normal stress test, healthy lifestyle, absence of cardiac medications, no peripheral vascular symptoms, no carotid bruits, normal cardiac biomarkers. DIETSUPP-2MOS: Associated with meeting the criterion: supplementation, vitamins, minerals, herbal, nutrients, omega-3, probiotics, amino acids, antioxidants, enzymes, fiber supplements, protein powders, dietary intake, nutritional support, health regimen, daily intake, nutritional products, nutraceuticals, ingest, consumption, dietary habits. Associated with not meeting the criterion: no supplements, diet-only, food sources, natural intake, unaided nutrition, no additional nutrients, exclusive diet reliance, whole foods, supplement-free, no pills, no capsules, no powders, no artificial nutrients, no fortified products. KETO-1YR: Associated with the criterion being met: ketoacidosis, diagnosis, DKA, diabetic ketoacidosis, hyperglycemia, ketones, acidosis, hospitalization, medical records, lab results, blood tests, urine tests, pH imbalance, bicarbonate levels, anion gap, insulin deficiency, diabetes, emergency treatment, metabolic acidosis, high blood sugar, endocrinologist, medical history, past 12 months. Not associated with the criterion being met: no history, no episodes, stable blood glucose, normal ketone levels, euglycemia, balanced pH, absence of symptoms, no hospital admissions for DKA, controlled diabetes, effective insulin therapy, regular monitoring, no acid-base disturbances, no diabetic complications, no acute diabetes events, good glycemic control, no metabolic acidosis, within normal range lab values. ASP-FOR-MI: Associated with meeting the criterion of using aspirin/ASA to prevent myocardial infarction (MI) might include prophylaxis, cardiovascular risk, anticoagulant therapy, antiplatelet therapy, secondary prevention, primary prevention, low-dose aspirin, coronary artery disease (CAD), atherosclerosis, stroke prevention, and ischemic heart disease. Words indicating the criterion is not met could include aspirin allergy, contraindications, bleeding disorders, hemorrhagic stroke, peptic ulcer disease, anticoagulant use, drug interactions, non-compliance, and alternative therapies. HBA1C: For the criterion of an HbA1c value between 6.5% and 9.5%, associated words when the criterion is met might include "eligible," "controlled diabetes," "within range," "moderate hyperglycemia," "acceptable," "enrollment criteria satisfied," and "qualified." Conversely, words associated with the criterion not being met could include "ineligible," "exclusion," "below threshold," "non-diabetic," "above threshold," "poorly controlled diabetes," "severe hyperglycemia," "disqualified," and "out of range." These terms reflect whether a potential participant's HbA1c levels fall within the specified range for inclusion in the clinical trial. CREATININE: Associated with elevated serum creatinine or serum creatinine levels above the upper limit of normal: renal impairment, kidney dysfunction, nephrotoxicity, renal insufficiency, chronic kidney disease (CKD), acute kidney injury (AKI), glomerulonephritis, reduced glomerular filtration rate (GFR), proteinuria, hematuria, azotemia, uremia, nephropathy, hypertension, diabetes mellitus, dehydration, rhabdomyolysis, muscle mass increase, dietary intake (high meat consumption), certain medications (e.g., ACE inhibitors, NSAIDs, aminoglycosides), and supplements (e.g., creatine). Words associated with serum creatinine levels within or below the normal range: normal renal function, healthy kidneys, adequate glomerular filtration, absence of renal disease, stable kidney condition, normal hydration status, normal muscle mass, and balanced diet. MI-6MOS: Associated with meeting the criterion of a recent myocardial infarction (MI) or current MI: chest pain, angina, ECG changes, elevated troponins, ST elevation, Q waves, coronary angiography, revascularization, stent placement, coronary artery bypass grafting (CABG), thrombolysis, aspirin, beta-blockers, ACE inhibitors, shortness of breath, cardiac enzymes, heart attack, percutaneous coronary intervention (PCI), left ventricular dysfunction, echocardiogram abnormalities. Associated with not meeting the criterion: stable angina, normal ECG, normal troponin levels, no ST changes, no Q waves, no recent coronary intervention, no recent revascularization, no symptoms of heart failure, normal cardiac function, no recent chest pain, clear echocardiogram, absence of cardiac biomarkers, no history of MI in the past six months.

Common failures

When screening for date-sensitive criteria, we observed several failures in temporal reasoning. These failures often occurred when evidence was correctly retrieved and identified by the LLM, but a date was not correctly associated with it or reasoned about. For example, when screening a patient for MI-6MOS, the LLM often produces results as shown below. False negatives due to errors in temporal reasoning were also observed but were much rarer.

The patient had a myocardial infarction in the past six months, as indicated by the history of NSTEMI in March, 2136, and subsequent cardiac catheterization revealing in-stent stenosis in LCx stent, which was addressed with bare metal stent placement.

In some cases, the LLM correctly identifies evidence but makes an incorrect conclusion based on said evidence. These cases seem most common when screening complex criteria with multiple requirements, such as ADVANCED-CAD or MAJOR-DIABETES. These errors seem to be due to the LLM failing to understand the criteria requirements fully. In the case of ADVANCED-CAD, false positives are common due to the LLM identifying evidence that meets some criteria requirements but not a sufficient amount. An example of this type of error is shown below, where the LLM classifies a patient as meeting ADVANCED-CAD, but the ground truth rules that the patient does not meet the criteria.

The patient has a history of CAD (Coronary Artery Disease) as mentioned in the PMH (Past Medical History). Additionally, the patient is on multiple CAD-related medications, including Atenolol and Simvastatin, which are beta-blockers and statins, respectively, used in the management of CAD.

Failures across all criteria can be attributed to insufficient document retrieval or information extraction from the LLM. This type of failure is commonly associated with false negatives, as the LLM is not provided with relevant context from the patient record. In most instances of this failure, reasoning such as ”No evidence found in the EHR” is listed. This may cause generally lower sensitivity than specificity values across all models.

In our testing, we found that hallucinations of evidence comprised a sizable number of misclassifications when using GPT-3.5 Turbo and a much smaller number of misclassifications when using GPT-4. The LLM often cited evidence from the prompt or expert guidance, even when this information is not found in the patient documents. For example, several patients with varying HbA1c values were incorrectly classified as meeting the HBA1C criterion. The LLM often cited ”HbA1c 7.2%” as evidence in these cases, reflecting a portion of the manually created expert guidance.
